# The Low-Diversity Fecal Microbiota of the Critically Endangered Kākāpō Is Robust to Anthropogenic Dietary and Geographic Influences

**DOI:** 10.3389/fmicb.2017.02033

**Published:** 2017-10-20

**Authors:** Elena K. Perry, Andrew Digby, Michael W. Taylor

**Affiliations:** ^1^School of Biological Sciences, University of Auckland, Auckland, New Zealand; ^2^Kākāpō Recovery Programme, Department of Conservation, Invercargill, New Zealand; ^3^Maurice Wilkins Centre for Molecular Biodiscovery, University of Auckland, Auckland, New Zealand

**Keywords:** gut microbiota, microbial communities, microbial ecology, symbionts, avian

## Abstract

The critically endangered kākāpō, an herbivorous parrot endemic to New Zealand, is subject to intensive management to increase its population size. Key aspects of the management program include supplementary feeding and translocation of kākāpō between different predator-free islands to optimize the genetic composition of the breeding populations. While these practices have helped boost the kākāpō population, their impact on the kākāpō fecal microbiota is uncertain. Previous studies have found that the kākāpō possesses a low-diversity fecal microbiota, typically dominated by *Escherichia/Shigella* spp. However, the question of whether the low diversity of the kākāpō fecal microbiota is an inadvertent consequence of human interventions has yet to be investigated. To that end, we used high-throughput Illumina sequencing of 16S rRNA gene amplicons obtained from fecal material of 63 kākāpō representing different diets, islands, and ages. Remarkably, neither supplementary feeding nor geographic location were associated with significant differences in the overall fecal microbial community structures of adult kākāpō, suggesting that the kākāpō's low-diversity fecal microbiota is both inherent to this species and robust to these external influences.

## Introduction

The kākāpō (*Strigops habroptilus*) is a critically endangered herbivorous parrot endemic to New Zealand, notable for its flightlessness, large size (1.3–4 kg), and lek mating system (Powlesland et al., 2006). Considerable effort has been devoted to the conservation of this species, which was decimated in the last century by habitat destruction and introduced mammalian predators. The kākāpō is now confined to a small number of predator-free offshore islands, where it is intensively managed by the New Zealand Department of Conservation (NZDOC) (Clout, 2006).

Previous research has revealed key aspects of the kākāpō's genetics (Robertson, 2006), behavior (Powlesland et al., 2006), growth (Farrimond et al., 2006), habitat selection (Walsh et al., 2006), and diet (Best, 1984; Butler, 2006; Cottam et al., 2006). As a result, scientifically-informed management practices, including supplementary feeding and relocation among different islands, have helped the population increase from a low point of 51 birds in 1995 (Powlesland et al., 2006) to nearly 160 as of late 2016. In an attempt to further aid conservation efforts, kākāpō research interests have recently expanded to the gut and fecal microbiota (Waite et al., 2012, 2013, 2014). Avian gut microbiotas are shaped by diet (Rubio et al., 1998; Torok et al., 2008; Janczyk et al., 2009; Hammons et al., 2010; Waite and Taylor, 2014) and can impact host health in diverse ways ranging from increased energy harvest from food (Torok et al., 2011) to modulation of the host immune system (Crhanova et al., 2011). Hence, greater understanding of the influence of diet and geography on the kākāpō fecal microbiota may contribute to improved management practices and disease prevention.

Previous studies have found that the kākāpō hosts a low-diversity fecal microbiota typically dominated by *Escherichia/Shigella* (Waite et al., 2013, 2014). However, the potential effects of supplementary feeding and geographic relocation on the kākāpō fecal microbiota have yet to be examined. Given that the supplementary food is made from grains, legumes, and seeds, whereas the kākāpō's natural diet primarily consists of shoots, leaves, rhizomes, and (when available) podocarp fruit (Best, 1984; Cottam et al., 2006), we hypothesized that data generated from supplementally-fed birds might not reflect the natural state of the kākāpō fecal microbiota. In addition, the natural kākāpō diet may vary according to geographic differences in vegetation on different islands. We therefore also hypothesized that kākāpō adults and chicks living on different islands might exhibit divergent fecal microbiotas in ways that could be relevant to their health.

To test these hypotheses, we conducted a 16S rRNA gene survey on fecal samples from adult kākāpō on three different diets and three islands, as well as fecal samples from chicks on two islands. By determining the extent to which supplementary feeding and relocation may have disturbed the fecal microbiota of the kākāpō, we aimed to assess whether such practices may need to be recalibrated in order to promote the long-term health of this species. More generally, this work belongs to a growing movement to understand the microbiotas of critically endangered species and their importance to successful conservation efforts, as the relationship between animal-associated microbiotas and host health becomes increasingly apparent (Amato et al., 2013; Barelli et al., 2015).

## Materials and methods

### Sample collection

We obtained a total of 135 kākāpō fecal samples from 40 adults and 23 pre-fledged chicks (Table S1). Fresh fecal samples were collected from adult kākāpō on Maud Island (41° 1′ S, 173° 53′ E), Pearl Island (47° 11′ S, 167° 42′ E), and Whenua Hou/Codfish Island (hereafter referred to as Codfish Island) (46° 47′ S, 167° 38′ E) in the years 1998–2001, and primarily on Codfish Island in 2014–2016 (Figure S1). Fresh fecal samples from chicks were collected during the 2016 breeding season on Codfish Island and Anchor Island (45° 45′ S, 166° 31′ E). Codfish Island and Pearl Island harbor similar vegetation dominated by indigenous forests (Elliott et al., 2006), including podocarp trees such as rimu (*Dacrydium cupressinum*), miro (*Prumnopitys ferruginea*), and totara (*Podocarpus totara*) (Whitehead, 2007). In contrast, Maud Island contains pastures and a non-native pine plantation, along with some indigenous forest but no podocarps (Walsh et al., 2006). Anchor Island harbors beech (*Nothofagus* spp.), yellow silver pine (*Lepidothamnus intermedius*), and pink pine (*Halocarpus biformis*) in addition to the podocarp species found on Codfish Island (Department of Conservation, 2013). While a previous study (Waite et al., 2012) also included swab samples from the crop and choana of chicks, such samples are more difficult to access in adult kākāpō, due to both anatomical differences between chicks and adults and the difficulty of safely restraining adults. Therefore, we did not pursue collection of crop or choana samples in this study in order to have a more consistent basis for comparison across chicks and adults.

Fecal samples were collected as directly as possible from the birds into sterile polypropylene vials or clean re-sealable plastic zipper storage bags during routine health screenings and stored at −20°C. We avoided samples that dropped onto the ground or touched surfaces other than a clean bag before being transferred to the vials. Freezing at −20°C was done either within 1–4 h of collection in the case of adult samples, or the morning after overnight sample collection in the case of chick samples. Samples were shipped to the University of Auckland on dry ice and stored at −20°C thereafter. The longer time between sample collection and freezing for chick samples was unavoidable, as chicks could not be left unattended at night.

The fecal sample collection in this study was carried out as part of standard management of kākāpō, as approved and authorized by NZDOC. This non-invasive sampling does not require ethics approval from the NZDOC Animal Ethics Committee, which upholds NZDOC's obligations under the New Zealand Animal Welfare Act.

### Supplementary feeding

A subset of adult kākāpō on Codfish Island were provided with supplementary food prior to and during the 2014 and 2016 breeding seasons. Supplementary feeding began in the spring (October 2013 and October 2015) and continued through the following summer. Different populations separated by a valley which the birds rarely cross were fed either Harrison's High Potency Coarse (“HPC”) pellets or pumpkin-based (“Pumpkin”) pellets. HPC pellets were obtained from Harrison's Bird Foods and consisted of sunflower seeds, a variety of legumes and grains, sea kelp, and algae meal, with additional vitamins and minerals. Pumpkin pellets were manufactured by Wombaroo/Passwell Food Products and consisted primarily of white rice, yellow peas, pumpkin powder, linseeds, and tapioca starch. Pumpkin pellets were designed to mimic the protein-calcium ratio of rimu fruit, the preferred natural food of kākāpō on Codfish Island during breeding seasons (Cottam et al., 2006; Raubenheimer and Simpson, 2006).

Supplementary food was provided at feeding stations consisting of lidded plastic containers mounted on platforms. Each feeding station was placed within the home range of a different individual. Every adult kākāpō is outfitted with a radio transmitter that emits a unique frequency to enable tracking. Moreover, most feeding stations were programmed to unlock only upon detecting the radio signal of a specific individual. Together, these measures minimized opportunities for crossover between kākāpō on different diets. Supplementary food consumption was recorded for individuals every 4 days, and varied between 35 and 375 g per bird over that time period. Samples were collected at least 2 weeks after beginning the supplementary feeding program for a given individual.

### DNA extraction

DNA was extracted from frozen fecal samples using a variation on a previously described bead-beating method (Waite et al., 2012). The original method was modified to improve the removal of PCR inhibitors such as polyphenols and polysaccharides, which are prevalent in kākāpō fecal samples due to their herbivorous diet. One hundred milligrams of each sample was washed twice in 70% ethanol. Washed samples were resuspended in 1 mL of extraction buffer [100 mM Tris-HCl (pH 8), 20 mM EDTA, 100 mM sodium phosphate (pH 8), 1.5 M NaCl, 2% CTAB] with 30 mg of acid-washed polyvinylpolypyrrolidone (PVPP) and 200 mg of 0.1 mm zirconia/silica beads in a 2 mL cryotube. Samples were agitated in a FastPrep FP120 bead beater at 5.5 ms^−1^ for 30 s, followed by incubation at 65°C for 30 min, with mixing by inversion every 10 min. Samples were briefly cooled on ice, then combined with 500 μL of 24:1 chloroform/isoamyl alcohol and mixed by inversion. Following centrifugation at 13,000 rpm for 10 min, the supernatant was transferred to a fresh tube. DNA was precipitated by adding 0.6 vol isopropanol and 0.1 vol sodium acetate (3 M, pH 5.2), mixing thoroughly, and incubating overnight at −20°C. The next day, samples were centrifuged at 13,000 rpm at 4°C for 30 min. The supernatant was removed and the DNA pellet was washed twice with ice-cold 70% ethanol.

To further purify the extracted DNA, the pellet was dissolved in 20 μL of TE buffer, followed by the addition of 30 mg acid-washed PVPP and 800 μL high-salt TE (containing 1.5 M NaCl). The mixture was vortexed at 1,400 rpm for 5 s. To the same tube, 200 μL of pre-warmed (65°C) NaCl/CTAB solution (0.7 M and 10%, respectively) was added, followed by a 30 min incubation at 65°C, with mixing by inversion every 10 min. The samples were then extracted twice with 500 μL of 24:1 chloroform/isoamyl alcohol. After the second extraction and centrifugation at 13,000 rpm for 10 min, the supernatant was centrifuged at 13,000 rpm for 2 min to pellet any remaining PVPP. The supernatant was then precipitated with isopropanol and sodium acetate overnight at −20°C. Following centrifugation and washing, the final DNA pellet was air dried, resuspended in 20 μL of 10 mM Tris-HCl (pH 8), and stored at 4°C (< 1 month until PCR amplification) or −20°C (long-term).

### PCR amplification and illumina amplicon sequencing

The variable V3-V4 region of the bacterial 16S rRNA gene was targeted for PCR amplification using primers synthesized with Illumina adapter overhang sequences (341F: 5′-TCGTCGGCAGCGTCAGATGTGTATAAGAGACAG**CCTACGGGNGGCWGCAG**−3′; 806R: 5′-GTCTCGTGGGCTCGGAGATGTGTATAAGAGACAG**GACTACHVGGGTATCTAATCC**−3′; bold text indicates the sequences that bind to the 16S rRNA gene) (Klindworth et al., 2013). Each reaction contained 12.5 μL 2X KAPA Plant PCR Buffer (Kapa Biosystems), 0.75 μL 10 μM 341F primer, 0.75 μL 10 μM 806R primer, 0.2 μL KAPA3G Plant DNA Polymerase (Kapa Biosystems), 9.8 μL UltraPure water (Invitrogen), and 1 μL template DNA. A negative control with UltraPure water was also included for each set of reactions. Cycling conditions were as follows: initial denaturation at 95°C for 3 min, 30 (Anchor Island samples) or 35 (all other samples) cycles of denaturation at 95°C for 20 s each, annealing at 57°C for 15 s, and extension at 72°C for 30 s, followed by a final elongation step at 72°C for 1 min. PCR was performed in triplicate and the reactions were pooled for each sample. Amplicon size and negative controls were validated by visualizing the PCR products on a 1% agarose gel with SYBR Safe DNA Gel Stain (Invitrogen).

PCR products were purified using AMPure XP beads (Beckman Coulter), quantified using the High Sensitivity (HS) dsDNA kit on a Qubit® Fluorometer 1.0 (Invitrogen), and diluted in UltraPure water to a final concentration of 5 ng/μL. Five samples that were less than 5 ng/μL were not diluted. Amplicon size was validated for a random selection of samples using a Bioanalyzer DNA 1000 chip (Agilent Technologies, Inc.). Libraries were prepared by New Zealand Genomics Limited, followed by sequencing on the Illumina MiSeq 2x300 platform. Sequence data were submitted to the NCBI Sequence Read Archive under the accession number SRP096634.

### Sequence data processing

Paired reads were merged in USEARCH (v7.0.1090; Edgar, 2013a), truncating reads at the first position with Q < 3 and discarding merged reads shorter than 200 bp. In addition, merged reads were discarded if the expected number of errors was > 1 (Edgar and Flyvbjerg, 2015). The remaining reads were clustered into operational taxonomic units (OTUs) at a threshold of 97% sequence identity using the UPARSE-OTU algorithm, which includes built-in chimera checking (Edgar, 2013b). All subsequent steps of sequence processing were performed in QIIME 1.8.0 (Caporaso et al., 2010b). Following alignment of the representative sequences from each OTU against the Greengenes reference alignment version 13_8 (DeSantis et al., 2006) with PyNAST (Caporaso et al., 2010a), the representative sequences were assigned taxonomic classifications by calling the mothur naïve Bayesian classifier (Wang et al., 2007; Schloss et al., 2009) and the RDP v14 reference taxonomy provided on the mothur wiki (www.mothur.org/wiki/RDP_reference_files). OTUs identified as chloroplasts, mitochondria, or archaea (collectively totaling 130,488 reads) were removed from the data set, leaving a final total of 6,452,576 reads. FastTree was used to construct a phylogenetic tree (Price et al., 2009), from which weighted and unweighted UniFrac distance matrices were generated following rarefaction of the data set to 4,275 sequences per sample.

### Statistical analysis

Permutational multivariate analysis of variance (PERMANOVA) statistical analyses of UniFrac distance matrices were performed in PERMANOVA+ in PRIMER v6 software (Anderson et al., 2008), using type III sums of squares with 4,999 unrestricted permutations of the raw data. We used a nested design for the diet comparison, the comparison of adults and chicks on Codfish Island, and the comparison of chicks from different islands: samples were nested within individuals, which were in turn nested within diet, island, or age. The individual from which the sample was drawn was treated as a random effect, whereas diet, island, and age were considered fixed effects. For the analysis of birds that were relocated to Codfish from different islands, we used an incomplete crossed design (individual x island). We tested for homogeneity of dispersions using permutational analysis of multivariate dispersions (PERMDISP) in PERMANOVA+ with 4,999 permutations (Anderson et al., 2008). To verify that significant PERMANOVA results were not an artifact of pseudoreplication, we conducted 1000 trials in which we randomly subsampled down to one sample per individual, followed by PERMANOVA with the function “Adonis” in the R package vegan (version 2.4-1; Oksanen et al., 2016).

Non-metric multidimensional scaling (nMDS) ordinations were generated in R with the package vegan and visualized using the package ggplot2 (version 2.1.0; Wickham, 2009). We performed linear discriminant analysis effect size (LEfSe) analysis (Segata et al., 2011) via the online Galaxy platform (Afgan et al., 2016). We used the one-against-all strategy for multi-class analysis in LEfSe, i.e., an OTU would be identified as a marker OTU if its abundance differed significantly in at least one of the classes relative to the rest, rather than requiring all pairwise comparisons between classes to be significant. We considered an OTU to be a marker OTU if the Kruskal-Wallis *p*-value was less than 0.05 and logarithmic linear discriminant analysis (LDA) score was greater than 2.0.

Alpha diversity metrics (Chao1 and Simpson's evenness) were compared across conditions using generalized linear mixed-effects models in R. We chose to compare species richness (Chao1) separately from species evenness (Simpson's evenness), rather than using a combined diversity index such as the Shannon diversity index or Simpson's diversity index, because such combined indices can obscure important differences: two communities could differ in both richness and evenness, yet have a similar Shannon or Simpson's diversity index. We used the R package MASS (version 7.3.45; Venables and Ripley, 2002) to perform regressions and the package MuMIn (version 1.15.6; Barton, 2016) to calculate pseudo-*R*^2^. For all comparisons, the individual from which the sample was drawn was included as a random effect to account for pseudoreplication. The fixed effects were diet, island, or age. For the Chao1 species richness metric, we employed quasi-Poisson regression as an alternative to Poisson regression for overdispersed count data (Crawley, 2005), while for Simpson's evenness, we performed Gaussian linear regression.

## Results and discussion

### General characteristics of the kākāpō fecal microbiota

**Figure 1:**
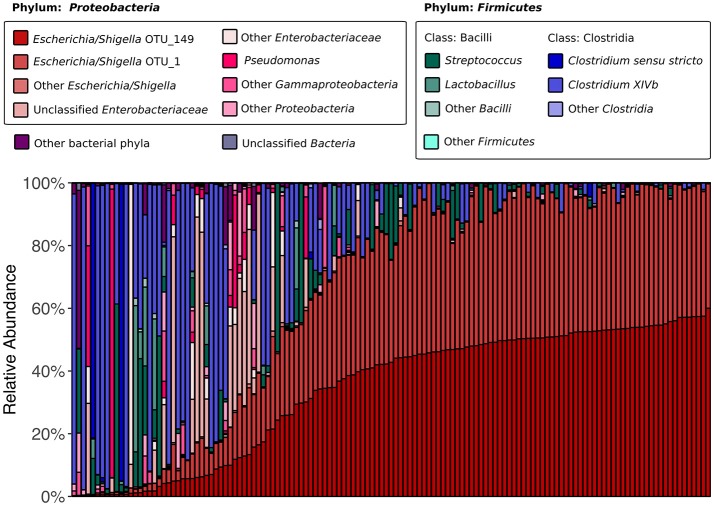
16S rRNA gene-based fecal microbiota composition of the kākāpō population. All 135 samples are arranged along the x-axis from lowest to highest relative abundance of *Escherichia/Shigella* OTU_149 (the most abundant OTU overall). OTU_149 and OTU_1 are the two OTUs that were present in all samples. The six genera that each accounted for >1% of total sequences were *Escherichia/Shigella, Pseudomonas, Streptococcus, Lactobacillus, Clostridium sensu stricto*, and *Clostridium XIVb*; each is represented by a different color. Unclassified *Enterobacteriaceae* also accounted for >1% of total sequences.

We began by assessing the distributions of major taxa and alpha diversity across all collected samples. 16S rRNA gene sequencing revealed that the kākāpō fecal microbiota is dominated by members of the bacterial phyla *Proteobacteria* and *Firmicutes* (75.5 and 23.5% of all sequences respectively; Figure 1), in line with previous studies (Waite et al., 2012, 2013, 2014). Good's coverage was >0.96 for all samples, indicating that the majority of bacterial diversity had been captured. Alpha diversity metrics were somewhat higher than in Waite et al. (2014), with a mean Shannon diversity index of 1.98, Simpson's diversity index of 0.63, and Chao1 richness of 82.5, as compared to 0.95, 0.58, and 60.5 respectively, which may reflect the greater sampling depth. Nevertheless, these alpha diversity metrics remain lower than those reported for the fecal microbiota of other parrots such as budgerigars and cockatiels (Garcia-Mazcorro et al., 2017), as well as more distantly related avian species such as the emu (Bennett et al., 2013), chicken (Hou et al., 2016), hoatzin (Godoy-Vitorino et al., 2008), and various penguins (Dewar et al., 2013). Simpson's evenness was also low in our samples, with a mean value of 0.12, indicating that most samples were dominated by relatively few OTUs. Two OTUs identified as *Escherichia/Shigella* appeared in all samples. These were also the only OTUs to appear in >90% of samples. Intriguingly, the abundances of these two OTUs were highly correlated (Pearson's *r* = 0.96, *p* < 0.001), which could suggest that they share a cooperative relationship or similar ecological niches. Alternatively, it could be that the same *Escherichia/Shigella* genome possesses two divergent copies of the 16S rRNA gene (Acinas et al., 2004). Taken together, these results reinforce previous findings that the kākāpō possesses an unusually low-diversity fecal microbiota of which *Escherichia/Shigella* is a characteristic member (Waite et al., 2014).

### Supplementary feeding effects

To evaluate the effects of different diets on the kākāpō fecal microbiota, we analyzed samples from adult kākāpō on Codfish Island that had been supplementally fed either HPC pellets (*n* = 20 from 9 birds) or Pumpkin pellets (*n* = 17 from 11) for at least 2 weeks prior to sample collection during the 2016 breeding season. We also included individuals that did not receive supplementary food (“Unfed”, *n* = 11 from 9), whose samples were collected during the same time period as those of the supplementally fed birds. Additional samples from supplementarily fed adults were available from the 2014 breeding season. However, because samples collected that season were almost exclusively from birds fed HPC pellets, and the kākāpō fecal microbiota can vary within individuals from year to year (Waite et al., 2014), we restricted our analysis of dietary effects to samples collected between November 2015 and March 2016.

**Figure 2:**
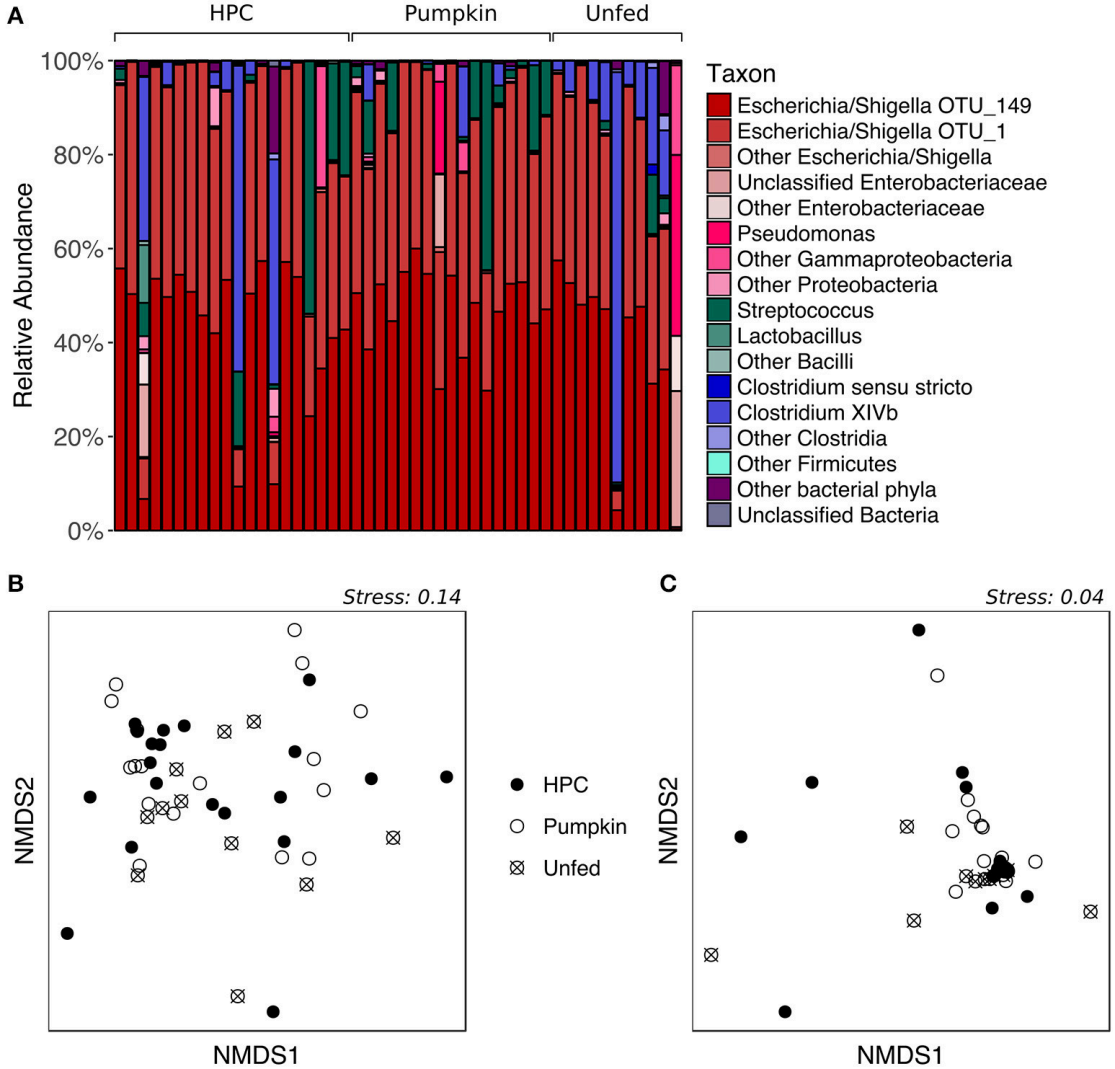
Supplementary feeding effects on the adult kākāpō fecal microbiota. **(A)** Relative abundances of different taxa for adults on three different diets. HPC, Harrison's High Potency Coarse pellets; Pumpkin, pumpkin-based pellets; Unfed, no history of supplementary feeding in the 2016 breeding season prior to sampling. Samples within the HPC and Pumpkin groups were arranged from left to right in decreasing order of the amount of supplementary food consumed over the 2 weeks preceding sample collection. Samples within the Unfed group were arranged in alphabetical order by bird name. **(B)** nMDS ordination based on unweighted UniFrac distances. **(C)** nMDS ordination based on weighted UniFrac distances.

While the proportions of major taxa in the fecal microbiota varied considerably among individuals (Figure 2A), PERMANOVA of both unweighted and weighted UniFrac distances, which represent the phylogenetic distances between all sample pairs, revealed no significant effect of diet on fecal bacterial community structure (*p* > 0.05, Table S2). Similarly, nMDS ordination revealed no obvious patterns related to diet, as the samples did not form separate clusters on the basis of diet classification (Figures 2B,C). HPC samples appeared to have the greatest dispersion, potentially implying greater inter-individual variation than within other diet groups; however, testing for homogeneity of dispersions with PERMDISP indicated that this difference was not significant (*p* > 0.05, Table S3). The Chao1 species richness metric and Simpson's evenness were similar for all diet groups (*p* > 0.05 for Pumpkin and Unfed referenced to HPC; Table S4, Figure S2). However, despite the lack of a significant difference in overall community structure between birds on different diets, LEfSe analysis indicated that one OTU—OTU_156 (*Clostridium XIVb*)—tended to be present in greater abundance in Unfed birds compared to birds on HPC or Pumpkin, although it was also highly abundant in a few birds on HPC (*p* = 0.022, LDA score = 2.214; Figure S3).

Overall, these analyses suggest that, contrary to our initial hypothesis, supplementary feeding does not drive major changes in the kākāpō fecal microbiota. This result contrasts with previous studies in which dietary supplements significantly affected the bacterial community composition of avian gut microbiotas (Rubio et al., 1998; Torok et al., 2008; Janczyk et al., 2009). However, those studies were carried out in domestic chickens, allowing for a more tightly controlled experimental setup than is possible with a wild, free-living bird. Moreover, studies on the gut microbiota of domestic chickens are routinely based on samples of intestinal tissue and gut contents rather than fecal material. Unlike those studies, we cannot examine whether changes occurred elsewhere in the kākāpō gut microbiota that were not reflected in the fecal microbiota, as destructive sampling to obtain intestinal tissue or contents is not possible in such a critically endangered species.

We note that while individual kākāpō varied in the quantity of supplementary food they consumed over the 2 weeks prior to sample collection, this variation was not visibly correlated with the proportions of major taxa in the fecal microbiota (Figure 2A). Together with the lack of a statistically significant difference between different diet groups in terms of overall community structure, this observation suggests that the inter-individual variation cannot be explained by the supplementary feeding regime. Unfortunately, to date, technical and logistical limitations have prevented accurate assessment of the types and quantities of natural foods consumed by individual kākāpō, which could potentially account for the observed variation in fecal microbiota composition. Genetic differences between individuals might also contribute to differences in their fecal microbiota, as has been demonstrated in other species (Banks et al., 2009; Hall et al., 2017). An effort is currently underway to sequence the genomes of all living kākāpō, which may help address this hypothesis.

Regardless of the variation among individuals within the same diet group, the apparent robustness of the overall community structure of kākāpō fecal microbiota to differences in supplementary feeding status is particularly interesting given its relatively low bacterial diversity. To our knowledge, the relationship between the diversity and stability of fecal microbiotas has not previously been investigated in birds; however, in humans, lower diversity of the fecal microbiota has been associated with decreased stability upon a change in dietary fiber intake (Tap et al., 2015). It is possible that the core members of the kākāpō fecal microbiota are more metabolically flexible than those of domestic chickens and humans and are therefore sufficient to utilize a variety of substrates. Indeed, Waite and Taylor (unpublished data) found that the dominant bacterial lineage in the kākāpō fecal microbiota (identified as *Escherichia fergusonii*) possesses genes for metabolizing both starch, which is a major component of the supplementary diets, and cellulose, which is the primary carbohydrate form in natural kākāpō foods (Toft and Wright, 2015). However, additional functional characterization of the enzymes encoded by these genes would be required to support this proposed explanation for the robustness of the kākāpō fecal microbiota to supplementary feeding.

### Age effects

**Figure 3:**
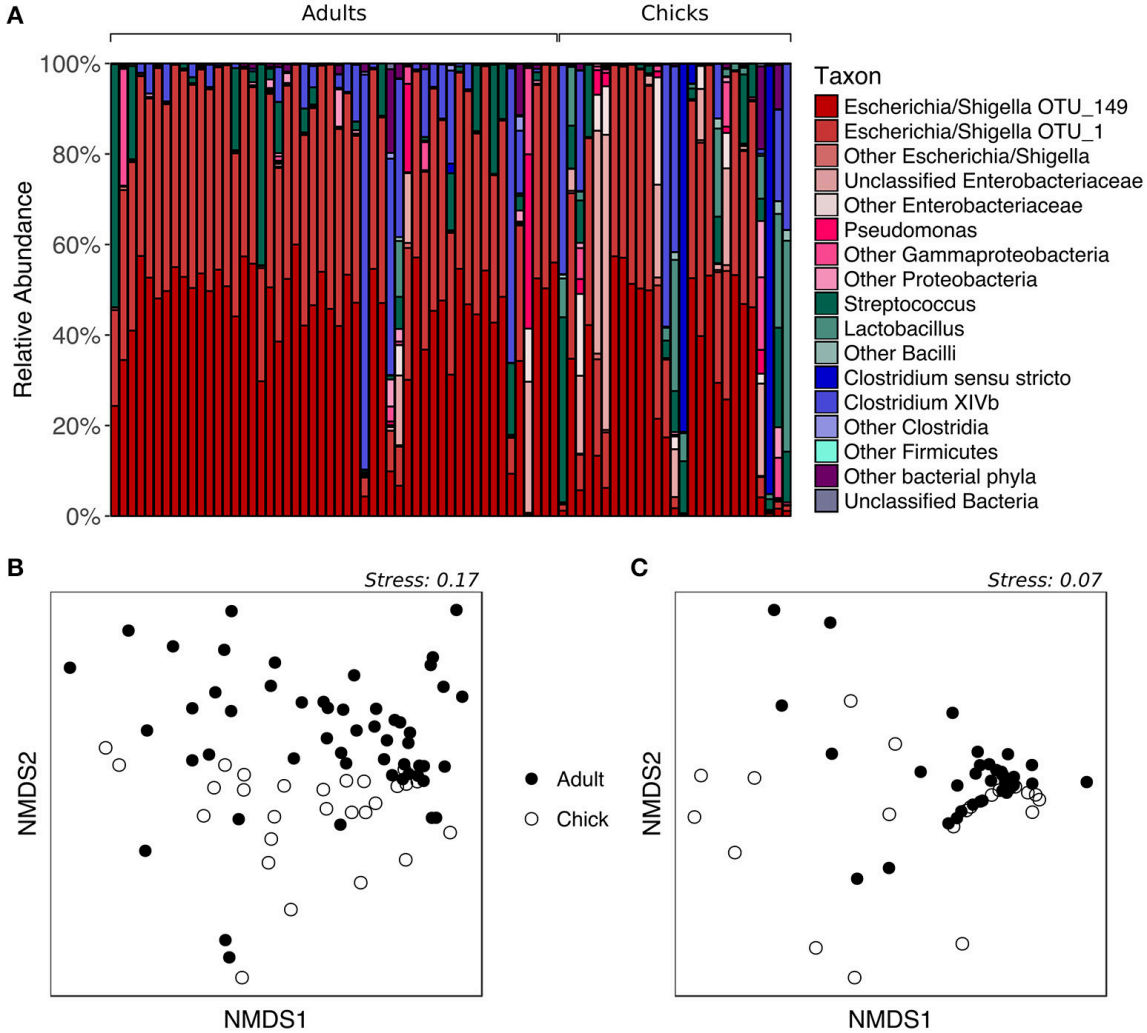
Fecal microbiota composition of chicks vs. adult kākāpō on Codfish Island. **(A)** Relative abundances of different taxa in chick and adult samples. Samples are grouped by age and arranged alphabetically by bird name within each age group. **(B)** nMDS ordination based on unweighted UniFrac distances. **(C)** nMDS ordination based on weighted UniFrac distances.

To determine whether fecal microbiota community structure differs between age groups, we analyzed samples collected from kākāpō adults and chicks on Codfish Island during the 2016 breeding season. All adult samples were included regardless of diet, given the above finding that supplementary feeding had no significant effect on the overall composition of the fecal microbiota. As expected, the majority of both adult and chick samples were dominated by *Escherichia/Shigella*, with a smaller number containing a high proportion of *Firmicutes* such as *Streptococcus, Lactobacillus*, or *Clostridia* (Figure 3A). PERMANOVA of unweighted UniFrac distances initially suggested a significant effect of age on the overall fecal microbiota community structure (*p* = 0.0152, Table S2). However, after randomly subsampling down to one sample per individual 1000 times, we found that a significant *p*-value (< 0.05) was retained in only 60.6% of PERMANOVA trials (Table S5). The nMDS plot for unweighted UniFrac is consistent with these mixed statistical results, as adults and chicks clustered largely but not entirely separately (Figure 3B). For weighted UniFrac distances, on the other hand, neither PERMANOVA (*p* > 0.05, Table S2) nor nMDS ordination (Figure 3C) indicated any difference between adults and chicks. Overall, these results suggest that adults and chicks tend to share similar dominant taxa, and that while there may be differences in the presence of rarer taxa, these differences are not consistent.

**Figure 4:**
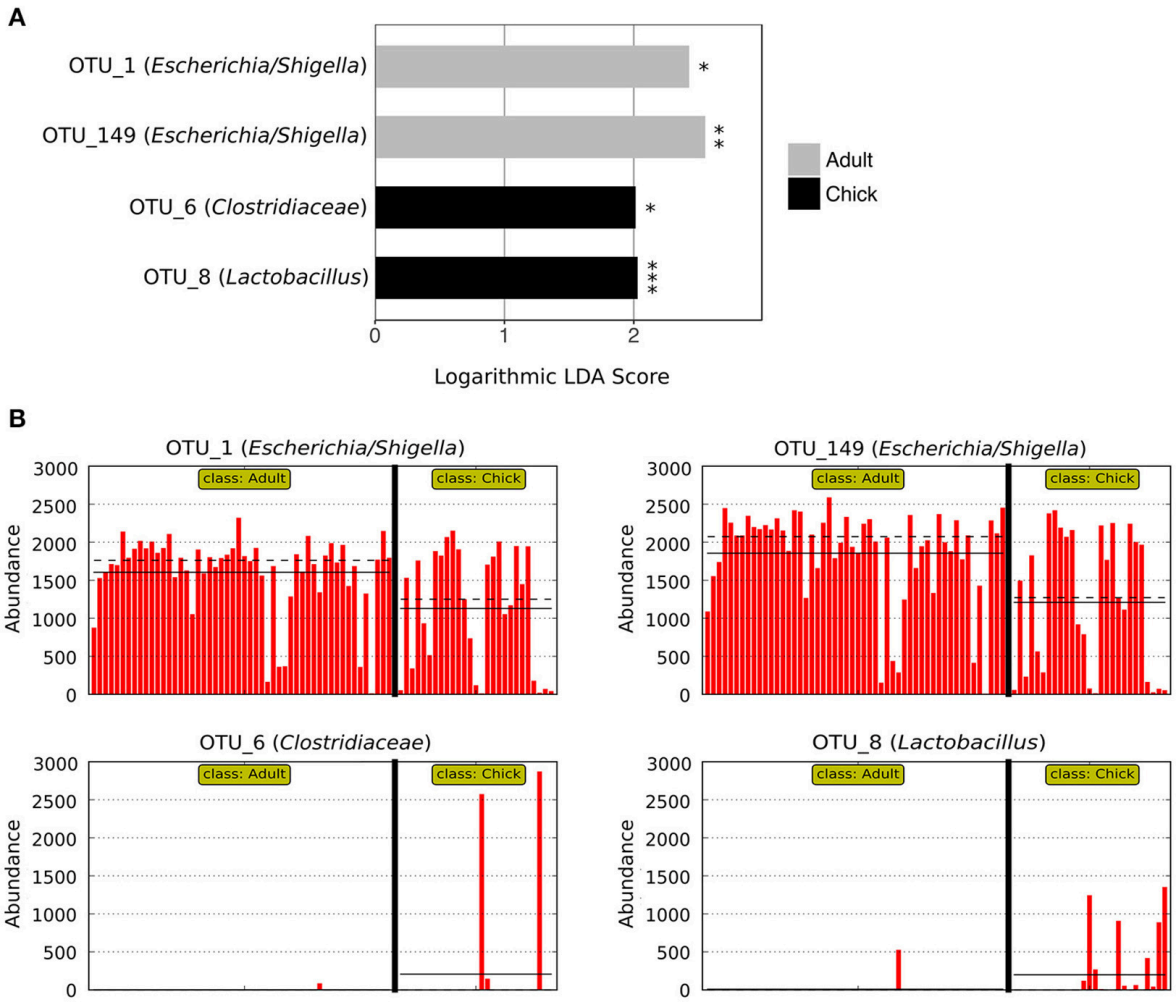
Marker OTUs for chicks vs. adult kākāpō on Codfish Island. **(A)** Marker OTUs were identified using the LEfSe (linear discriminant analysis effect size) method. OTUs were considered “markers” if two conditions were satisfied: (1) the Kruskal-Wallis *p*-value from the first step of LEfSe was <0.05 (^*^p < 0.05, ^**^p < 0.01, ^***^p < 0.001), and (2) the logarithmic linear discriminant analysis (LDA) score was >2.0. **(B)** Raw data for each marker OTU. Samples are arranged along the x-axis in each plot, separated by age. Solid horizontal lines represent the mean abundance of the OTU for each age group, while dashed horizontal lines represent the median.

LEfSe analysis revealed two marker OTUs for chicks, OTU_6 (*Clostridiaceae*) (*p* = 0.046, LDA score = 2.015) and OTU_8 (*Lactobacillus*) (*p* = 2.03 × 10^−6^, LDA score = 2.030), although these OTUs were not present in all chick samples (Figures 4A,B). Interestingly, LEfSe also revealed that even though OTU_1 (*Escherichia/Shigella*) and OTU_149 (*Escherichia/Shigella*) were highly prevalent and abundant in both adults and chicks, the relative abundances tended to be higher in adults (OTU_1: *p* = 0.026, LDA score = 2.428; OTU_149: *p* = 0.004, LDA score = 2.553; Figures 4A,B). Despite these differences, neither the Chao1 species richness metric nor Simpson's evenness differed significantly between adults and chicks (*p* > 0.05 for chicks referenced to adults; Table S4, Figure S2).

The increased prevalence and abundance of members of *Clostridiaceae* and *Lactobacillus* in the fecal microbiota of chicks is consistent with previous findings on Codfish Island (Waite et al., 2014). Species of *Lactobacillus* in particular commonly dominate the avian crop (Kierończyk et al., 2016); thus, one possible explanation for the higher abundance of *Lactobacillus* in the chick fecal microbiota is that kākāpō chicks feed on pre-masticated food that presumably has been stored in the mother's crop (Cottam et al., 2006). However, despite the differential abundance of particular OTUs in adults vs. chicks, previous studies found no significant differences between adults and chicks in terms of overall community structure of the fecal microbiota (Waite et al., 2012, 2014). While our results were mixed, and therefore not necessarily inconsistent with the earlier studies, one likely explanation for the lack of any statistically significant overall difference in those studies despite OTU-level differences is the much smaller sample sizes—three samples per age group in the 2012 study, and eight to 10 samples per age group in the 2014 study, compared to 52 adult samples and 27 chick samples in the present study.

### Location effects on adults

**Figure 5:**
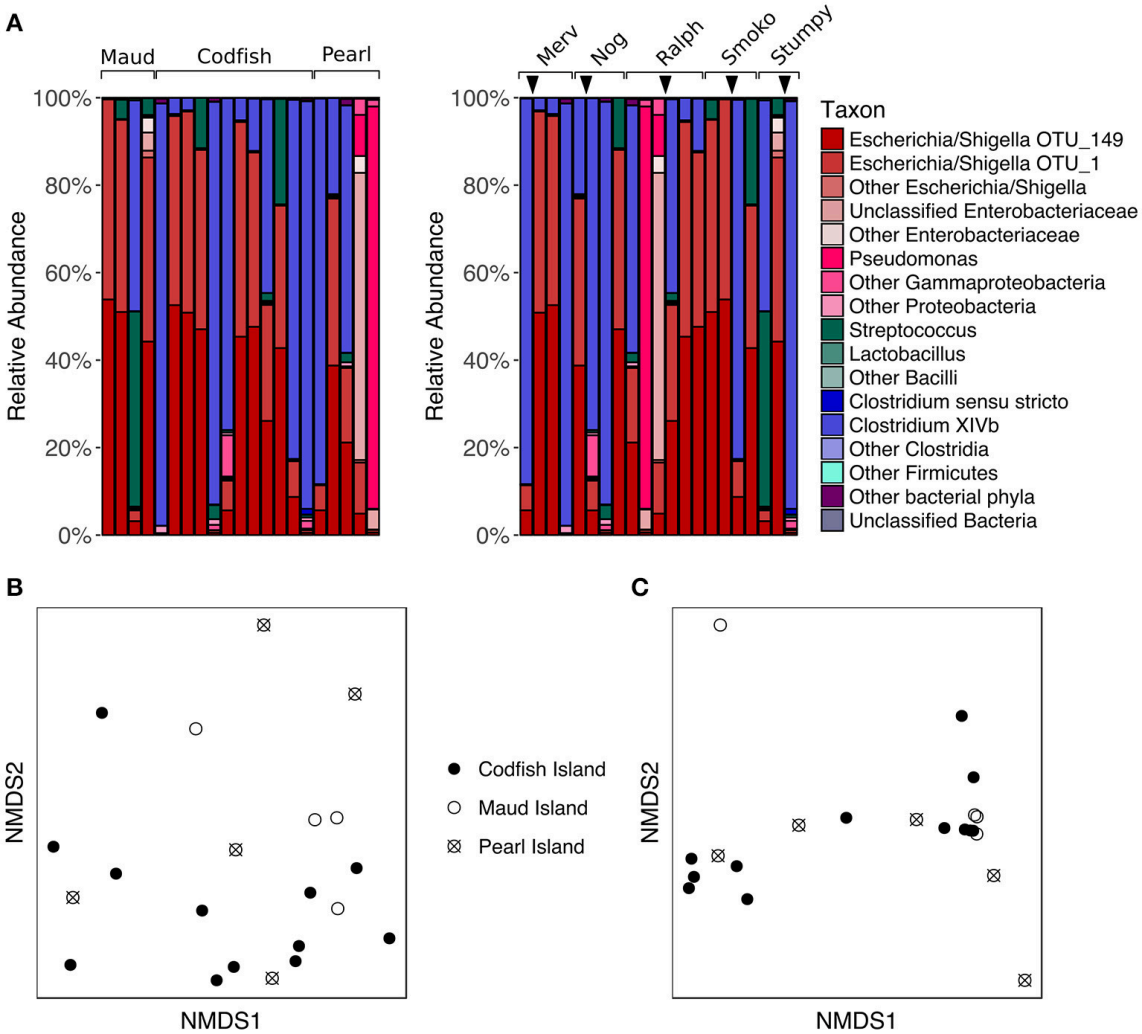
Effects of geographic relocation on the fecal microbiota of adult kākāpō. **(A)** Relative abundances of different taxa in samples from adults that were relocated from Maud Island or Pearl Island to Codfish Island. On the left, samples are grouped by island and arranged alphabetically by bird name within each island group. On the right, samples are grouped by individual and arranged from left to right in chronological order for each individual. Arrows indicate the time at which the individual was moved to Codfish Island. **(B)** nMDS ordination based on unweighted UniFrac distances. **(C)** nMDS ordination based on weighted UniFrac distances.

To assess whether different islands leave distinct signatures on the adult kākāpō fecal microbiota, we analyzed a set of five adult kākāpō for which we had samples collected both pre- and post-translocation to Codfish Island; three initially lived on Pearl Island, while the other two lived on Maud Island. We observed considerable variation in the proportions of major taxa within individuals over time (Figure 5A). However, PERMANOVA of unweighted and weighted UniFrac distances revealed no significant effect of island (*p* > 0.05, Table S2). Despite the small sample sizes, this result was not attributable to insufficient scope for permutation, as each PERMANOVA test reported >4,990 unique permutations. The nMDS plots (Figures 5B,C) and comparisons of the Chao1 species richness metric and Simpson's evenness likewise support the lack of a consistent biogeographical pattern (*p* > 0.05 for Maud and Pearl referenced to Codfish Island; Table S4, Figure S2).

The lack of a significant difference between adult samples collected on Pearl Island and Codfish Island was not entirely unexpected, as both islands share similar vegetation. Maud Island, on the other hand, possesses distinct vegetation characteristics and lacks podocarp trees (Walsh et al., 2006), which are a favored food source for kākāpō when available (Cottam et al., 2006). The failure of samples from Maud Island to cluster separately in nMDS ordination plots therefore suggests that even though the natural food availability differed on that island, the microbial communities in our samples were more strongly shaped by factors unrelated to geography. Similar to these results, studies of wild neotropical songbirds, hummingbirds, and insectivorous birds, as well as Adelie penguins, found little evidence of a relationship between geographic distance and fecal microbiota composition (Banks et al., 2009; Hird et al., 2015). This is in contrast to a study of wild mice, in which the authors found that geographic location was the most significant factor contributing to inter-individual variation in gut microbiota composition, which they suggested could be due to neutral dispersal limitation among microbes (Linnenbrink et al., 2013). Based on the lack of a geographic signature in our study, neutral dispersal limitation does not appear to explain a significant proportion of the variance among adult kākāpō fecal microbiotas, although this is not surprising given that many kākāpō have been relocated at least once.

### Location effects on chicks

**Figure 6:**
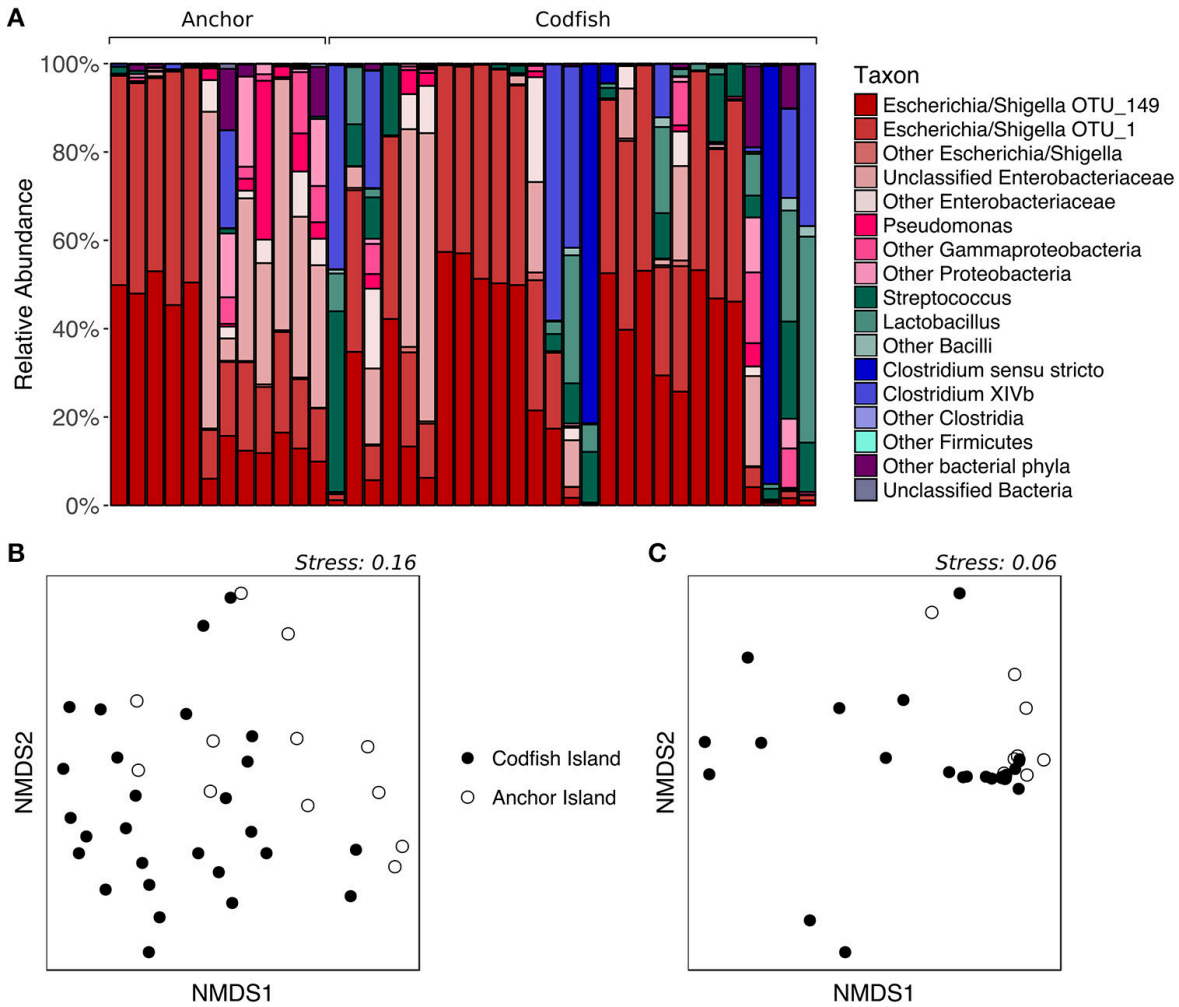
Effects of geographic location on the fecal microbiota of kākāpō chicks. **(A)** Relative abundances of different taxa in chick samples from Anchor Island or Codfish Island. Samples are grouped by island and arranged alphabetically by bird name within each island group. **(B)** nMDS ordination based on unweighted UniFrac distances. **(C)** nMDS ordination based on weighted UniFrac distances.

To determine whether kākāpō chicks hatched and raised on different islands possess divergent fecal microbiotas, we analyzed samples collected from chicks on Anchor Island and Codfish Island. The two groups visibly differed upon plotting the relative abundances of major taxonomic groups in their fecal microbiotas: representatives of the phylum *Firmicutes* were nearly absent from the Anchor Island samples, but accounted for a high proportion of sequences in several Codfish Island samples (Figure 6A). Indeed, in contrast to the results for adults on different islands, PERMANOVA of unweighted UniFrac distances for chicks indicated that samples collected on Anchor Island differed significantly from those collected on Codfish Island (*p* = 0.0022, Table S2), in accordance with the clustering apparent in the nMDS plot (Figure 6B). When we randomly subsampled down to one sample per individual 1,000 times to remove potential effects of pseudoreplication, PERMANOVA still returned a significant *p*-value (< 0.05) in 97.8% of the trials, which we interpret as reasonably strong evidence of a true difference in OTU membership between the two chick populations (Table S6).

**Figure 7:**
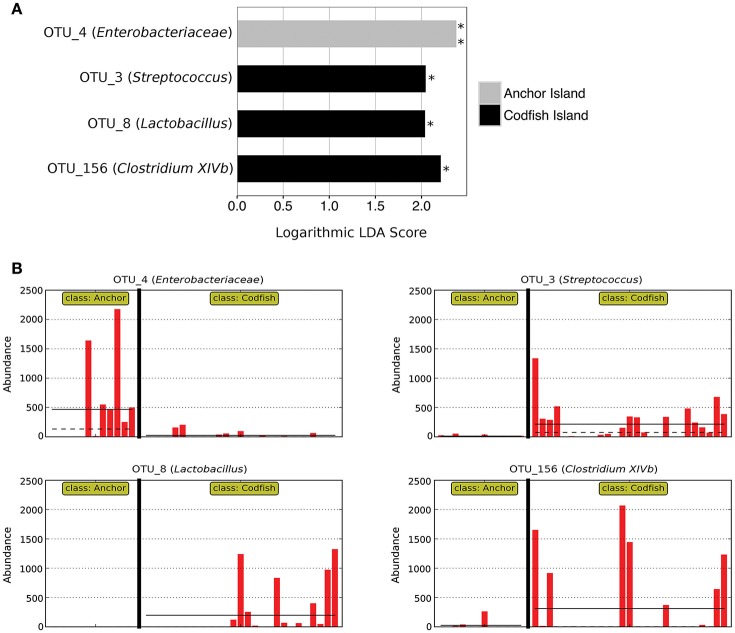
Marker OTUs for kākāpō chicks on Anchor Island vs. Codfish Island. **(A)** Marker OTUs were identified using the LEfSe (linear discriminant analysis effect size) method. OTUs were considered “markers” if two conditions were satisfied: (1) the Kruskal-Wallis *p*-value from the first step of LEfSe was < 0.05 (^*^p < 0.05, ^**^p < 0.01), and (2) the logarithmic linear discriminant analysis (LDA) score was >2.0. **(B)** Raw data for each marker OTU. Samples are arranged along the x-axis in each plot, separated by island. Solid horizontal lines represent the mean abundance of the OTU for each island group, while dashed horizontal lines represent the median.

In accordance with the differences apparent both from the PERMANOVA results and from Figure 6A, LEfSe analysis identified four marker OTUs that distinguish Anchor Island chicks from Codfish Island chicks. Anchor Island chicks were significantly enriched with OTU_4 (*Enterobacteriaceae*; *p* = 0.001, LDA score = 2.378), whereas Codfish Island chicks were enriched with OTU_3 (*Streptococcus*; *p* = 0.013, LDA score = 2.045), OTU_8 (*Lactobacillus*; *p* = 0.013, LDA score = 2.038), and OTU_156 (*Clostridium XIVb*; *p* = 0.032, LDA score = 2.209) (Figure 7). We also found that Anchor Island chicks had both significantly higher Chao1 species richness estimates (*p* = 0.044, pseudo-*R*^2^ = 0.0016) and significantly lower Simpson's evenness (*p* = 0.031, pseudo-*R*^2^ = 0.1373) than Codfish Island chicks (Table S4, Figure S2), although the low pseudo-*R*^2^ values indicate that location only accounted for a small proportion of the variance in these measures of alpha diversity.

In contrast to the above results, PERMANOVA of weighted UniFrac distances did not reveal a significant difference between chicks on Anchor Island vs. Codfish Island (*p* > 0.05), but this was surprising given the moderate clustering observed in the nMDS plot (Figure 6C). Importantly, PERMANOVA tends to lose power when used on unbalanced data sets in which the larger group has significantly greater dispersion (Anderson and Walsh, 2013). Using PERMDISP, we confirmed that the dispersions of samples from Codfish Island (*n* = 27, average deviation from group centroid = 0.167) were significantly larger than those of samples from Anchor Island (*n* = 12, average deviation from group centroid = 0.076) (*p* = 0.0254, Table S3). Thus, the failure to detect a significant difference based on weighted UniFrac distances may reflect the use of an overly conservative test. To our knowledge, alternatives to PERMANOVA that are robust in the face of unbalanced designs with heterogeneous dispersions have yet to be developed.

The differences we found in fecal microbiota composition for chicks on Anchor Island vs. Codfish Island may reflect the fact that the former were primarily fed natural foods by their mothers, including ripened rimu fruit, while the latter were primarily fed HPC or Pumpkin pellets as the rimu crop on Codfish failed to ripen. Given that we also found differences in fecal microbiota composition between adults and chicks on Codfish Island, these results may suggest that the fecal microbiota of kākāpō chicks is more sensitive than that of adults to dietary and/or other environmental influences related to geographic location. One explanation that would be consistent with these results is that kākāpō chicks have a more restricted diet than that of adults, being fed almost exclusively on rimu fruit where available, or otherwise on supplementary food pellets, whereas adults generally consume a more diverse range of foods throughout the year (Best, 1984; Cottam et al., 2006). Thus, it would not be surprising if the fecal microbiota of chicks is more strongly tied to geographic location, and hence diet. Additionally, kākāpō chicks may have a less well-developed immune system than adults, as has been demonstrated in other avian species (Killpack et al., 2013), which could lead to greater susceptibility to invasions of environmental bacteria; such bacteria could differ between islands. However, as we lacked sufficient adult samples from Anchor to compare with those of Anchor chicks and Codfish adults, it remains unclear whether the fecal microbiota of kākāpō chicks is indeed shaped more strongly by dietary and/or other environmental influences, or if Anchor Island possesses unique characteristics that similarly affect the adult fecal microbiota.

## Conclusion

Overall, our findings suggest that human interventions such as supplementary feeding and relocation to different islands do not significantly influence the overall community structure of the adult kākāpō fecal microbiota. As these practices have played a key role in increasing kākāpō breeding success rates in recent years, this work underscores the relevance to conservation biology of understanding the microbiotas of endangered species. While our data do not rule out potential effects of differences in natural food consumption or genetic background, this apparent robustness to human interventions is remarkable in light of the low diversity of the kākāpō fecal microbiota and the well-known effects of diet on other vertebrate fecal microbiotas. Future investigations of the metabolic capacity of the kākāpō fecal microbiota may help shed light on the mechanisms underlying its robustness to supplementary feeding and geographic location. It will also be important to follow up on the chicks that were hatched on Anchor Island and Codfish Island, in order to determine whether the differences present at an early age persist into adulthood. Nevertheless, it appears that both low diversity and a consistent presence of *Escherichia/Shigella* are true hallmarks of the natural kākāpō fecal microbiota.

## Author contributions

AD and MT conceived of and coordinated the study. EP performed the experiments and statistical analysis. EP, AD, and MT wrote the manuscript.

### Conflict of interest statement

The authors declare that the research was conducted in the absence of any commercial or financial relationships that could be construed as a potential conflict of interest.
