# Supplementary material for: The Low-Diversity Fecal Microbiota of the Critically Endangered Kākāpō Is Robust to Anthropogenic Dietary and Geographic Influences
Source: Front Microbiol. 2017 Oct 20;8:2033. doi: 10.3389/fmicb.2017.02033 (PMC5655120; doi:10.3389/fmicb.2017.02033)
Supplement: Supplementary file 7 [file Image1.PDF]

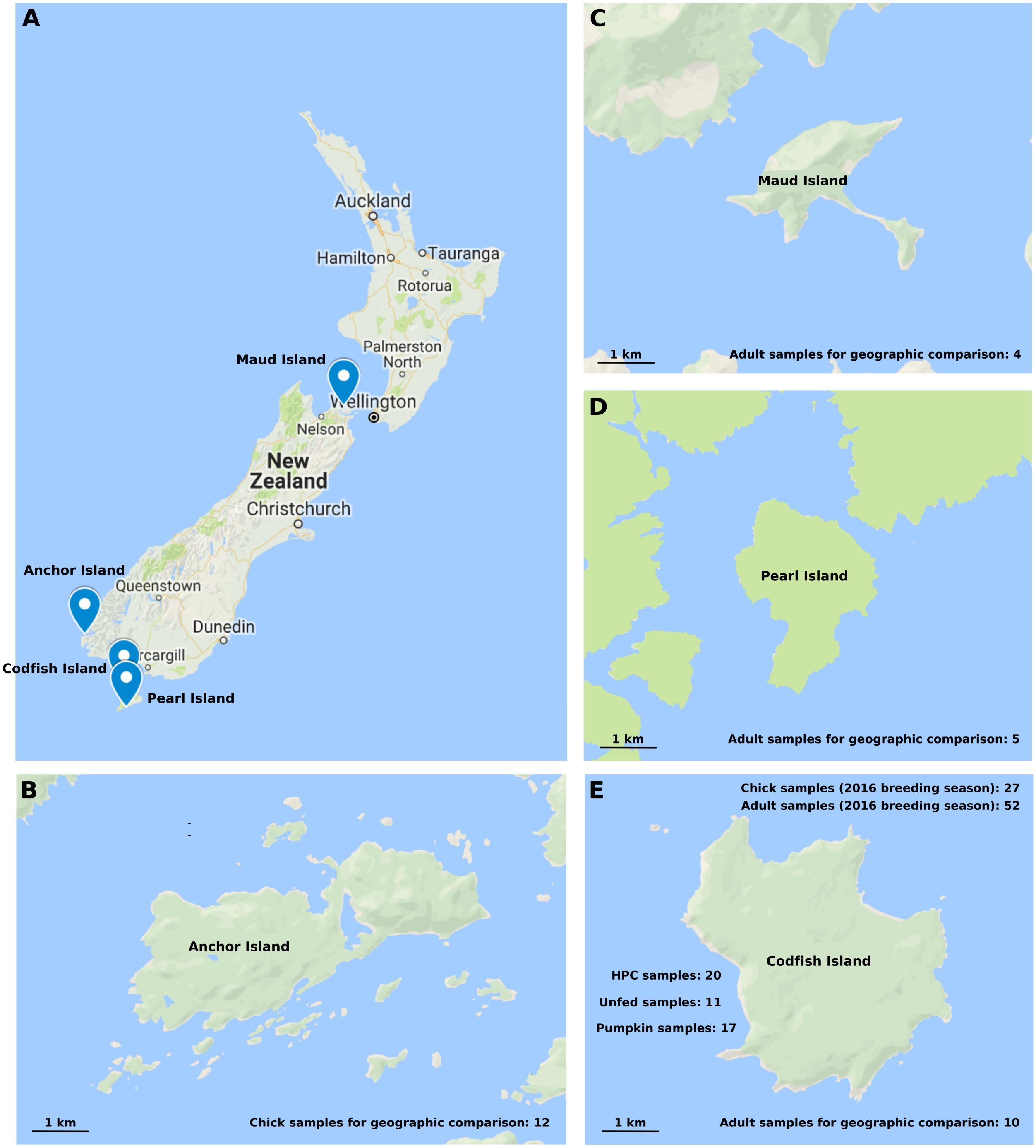

Figure S1. Map of islands showing where samples of different types were collected
Map data: Google. **(A)** Map of New Zealand showing the location of each island (blue markers). **(B, C, D, E)** Zoomed-in view of each island, with text indicating the type and number of samples collected from that island.
