# Supplementary material for: The Low-Diversity Fecal Microbiota of the Critically Endangered Kākāpō Is Robust to Anthropogenic Dietary and Geographic Influences
Source: Front Microbiol. 2017 Oct 20;8:2033. doi: 10.3389/fmicb.2017.02033 (PMC5655120; doi:10.3389/fmicb.2017.02033)
Supplement: Supplementary file 8 [file Image2.PDF]

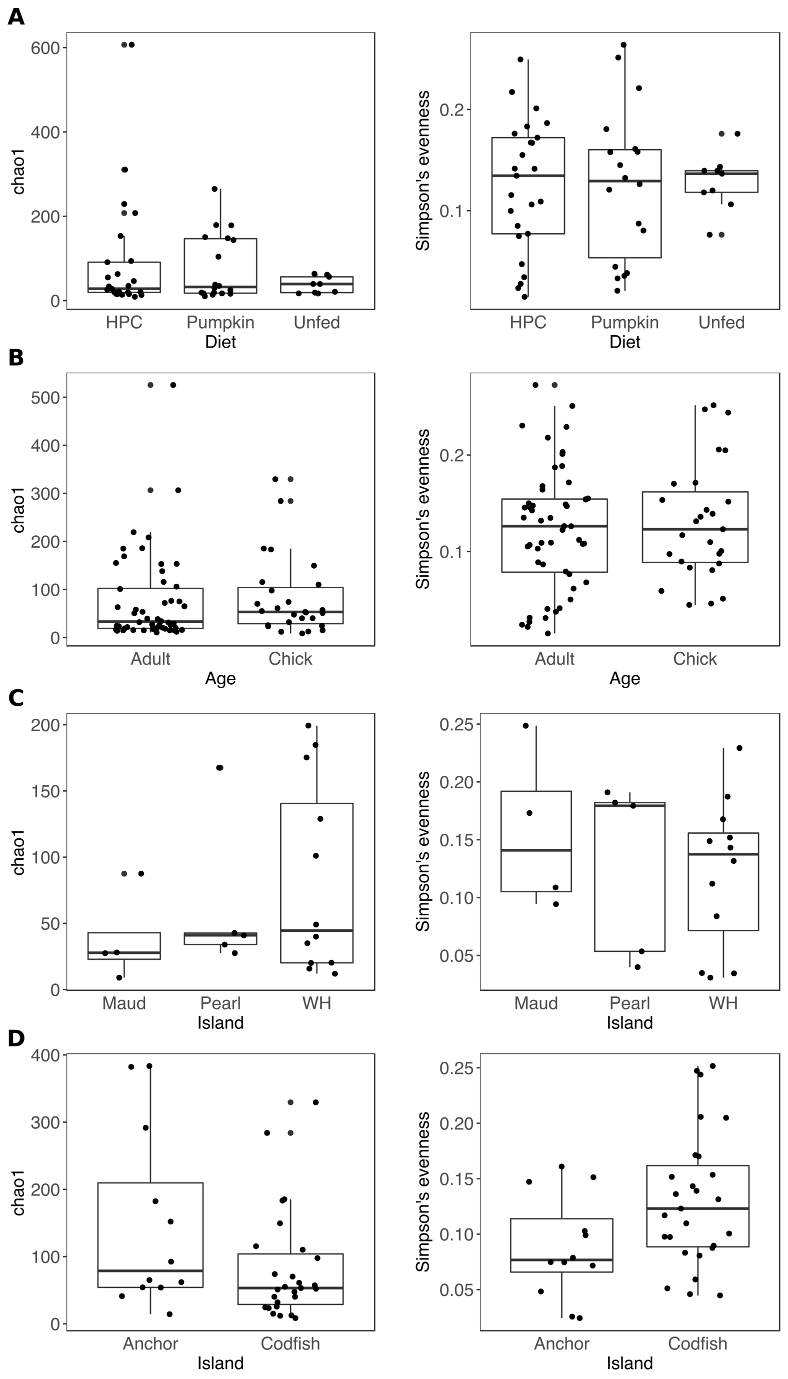

Figure S2. Alpha diversity of kākāpō on different supplementary diets, of different ages, or on different islands

Left: Chao1 species richness estimate. Right: Simpson's evenness. **(A)** Adult kākāpō on three different diets. HPC = Harrison's High Potency Coarse pellets. Pumpkin = pumpkin-based pellets. Unfed = no history of supplementary feeding in the 2016 breeding season prior to sampling. **(B)** Chicks versus adult kākāpō on Codfish Island during the 2016 breeding season. **(C)** Adults that were relocated from Maud Island or Pearl Island to Codfish Island. **(D)** Chicks that were hatched and raised on Anchor Island or Codfish Island.
