# Supplementary figures and images for: The Low-Diversity Fecal Microbiota of the Critically Endangered Kākāpō Is Robust to Anthropogenic Dietary and Geographic Influences
Source: Front Microbiol. 2017 Oct 20;8:2033. doi: 10.3389/fmicb.2017.02033 (PMC5655120; doi:10.3389/fmicb.2017.02033)

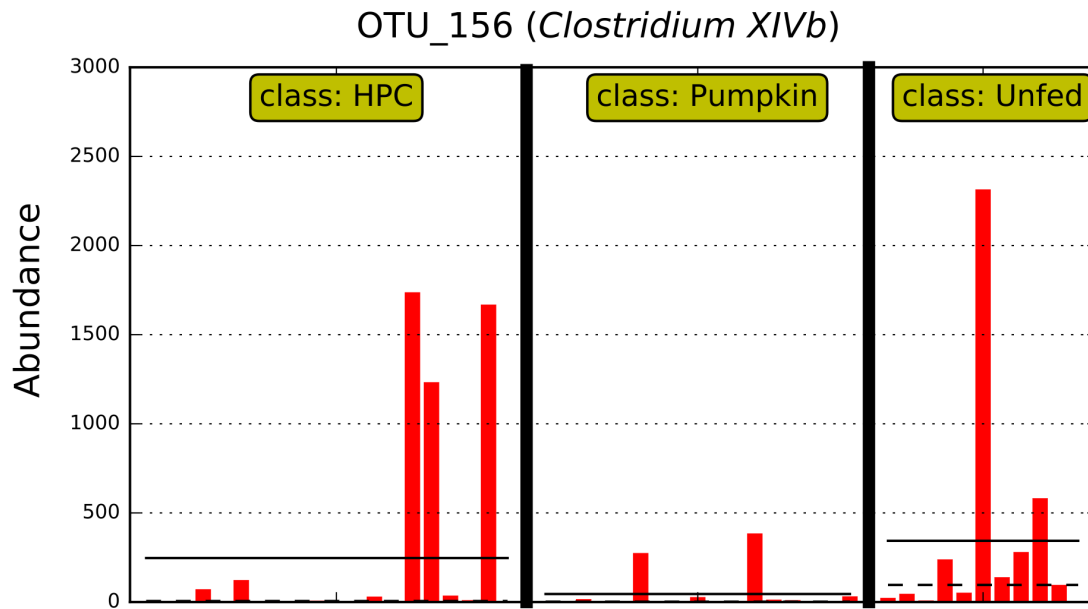

Supplement: Supplementary file 9 [file Image3.PDF]
